# MMP-2 and 9 in Chronic Kidney Disease

**DOI:** 10.3390/ijms18040776

**Published:** 2017-04-08

**Authors:** Zhengyuan Cheng, Manoj Hang Limbu, Zhi Wang, Jing Liu, Lei Liu, Xiaoyi Zhang, Pingsheng Chen, Bicheng Liu

**Affiliations:** 1 Department of Pathology and Pathophysiology, Medical School, Southeast University, Dingjiaqiao 87, Gulou District, Nanjing 210009, China; 230159257@seu.edu.cn (Z.C.); 223136077@seu.edu.cn (M.H.L.); 76wangzhi76@163.com (Z.W.); 230149691@seu.edu.cn (J.L.); 230169250@seu.edu.cn (L.L.); zhxynanjing@163.com (X.Z.); 2 Department of Nephrology, Zhongda Hospital, Southeast University, Dingjiaqiao 87, Gulou District, Nanjing 210009, China; liubc64@163.com

**Keywords:** gelatinases, matrix metalloproteinase, chronic kidney disease, non-proteolytic function

## Abstract

Gelatinases are members of the matrix metalloproteinase (MMPs) family; they play an important role in the degradation of the extracellular matrix (ECM). This effect is also crucial in the development and progression of chronic kidney disease (CKD). Its expression, as well as its activity regulation are closely related to the cell signaling pathways, hypoxia and cell membrane structural change. Gelatinases also can affect the development and progression of CKD through the various interactions with tumor necrosis factors (TNFs), monocyte chemoattractant proteins (MCPs), growth factors (GFs), oxidative stress (OS), and so on. Currently, their non-proteolytic function is a hot topic of research, which may also be associated with the progression of CKD. Therefore, with the in-depth understanding about the function of gelatinases, we can have a more specific and accurate understanding of their role in the human body.

## 1. Introduction

In recent years, with the increasing prevalence of chronic kidney disease (CKD), this disease has become a major public health problem in the world [1,2]. With the development of CKD, eventually, it will progress to end-stage renal disease (ESRD) [3] and leads to irreversible loss of renal function. In the early stages of CKD, the renal function is still in a compensatory period, but with the progression of the disease, the renal function will deteriorate, and finally, complete loss of renal function ensues. The main pathological changes include renal interstitial fibrosis, glomerulosclerosis, etc. [4], and renal interstitial fibrosis is considered to be the final outcome of all CKD regardless of its etiology [5]. The pathogenesis of renal interstitial fibrosis is complex, which is a combination of multiple factors. When kidney is injured, the occurrence of inflammation leads to infiltration of inflammatory cells, such as macrophages, lymphocytes, etc. In this process, the injured cells and the inflammatory cells in kidney release a large number of inflammatory mediators, such as [6,7,8] tumor necrosis factors (TNFs), monocyte chemoattractant proteins (MCPs), growth factors (GFs), etc., which on the one hand further aggravate the inflammatory reaction of the kidney and on the other hand result in activation of relevant cell signaling pathway, such as [9,10] transforming growth factor-β (TGF-β)/Smad and Notch, which promote the development of fibrosis. Eventually, this will lead to the extracellular matrix (ECM) deposition that will induce the blocking of the renal interstitial capillary bed and hypoxia. All of the above will promote the emergence of renal interstitial fibrosis; during this process, matrix metalloproteinases (MMPs), especially gelatinases, play a significant role.

The study of MMPs began in 1962 [11]; nowadays, there are 28 kinds of human-derived MMPs that have been discovered, which belong to the family of zinc-dependent endopeptidases. According to the structural and substrate specificity, MMPs can be divided into: collagenases, gelatinases, stromelysins, matrilysins, membrane-type MMPs and other types [12]. Gelatinases mainly include MMP-2 and MMP-9; its structure includes the signal peptide region [13], which mediates the nascent peptide chains to the cytoplasm and endoplasmic reticulum; the propeptide region keeps the gelatinases in an inactive form; the catalytic domain and the hemopexin domain (PEX), which connect each of them through a hinge structure; and PEX plays an important role in the regulation of substrate specificity and localization of MMP-2 and 9 [14].

## 2. The Regulation of Matrix Metalloproteinase-2 (MMP-2) and Matrix Metalloproteinase-9 (MMP-9) Expression

**Figure 1:**
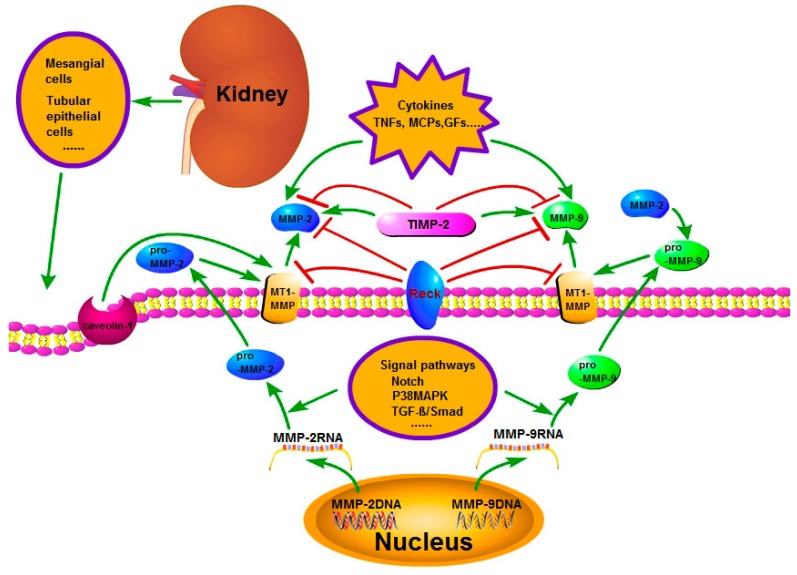
The regulation of activity and expression of matrix metalloproteinase-2 and 9 (MMP-2 and 9). The regulation mechanism is complicated. The signal pathways, such as Notch, P38MAPK and transforming growth factor-β (TGF-β)/Smad, regulate the production of pro-MMP-2 and pro-MMP-9. Tissue inhibitor of metalloproteinase-2 (TIMP-2) and membrane-type 1 matrix metalloproteinase (MT1-MMP) play an important role in the activation of pro-MMP-2 and pro-MMP-9 to MMP-2 and MMP-9. Additionally, MMP-2 also can promote the pro-MMP-9 to MMP-9. Reversion-inducing cysteine-rich protein with kazal motifs (RECK), TIMP-2, endocytosis and cytokines play an important part in the activity regulation of MMP-2 and MMP-9.

Under the normal condition, the mesangial cells, tubular epithelial cells, etc., in the human kidney can produce MMP-2 and 9, but always at a low level. However during the process of renal fibrosis, the mRNA transcription levels of MMP-2 and 9 are upregulated rapidly due to the abnormal activation and interactions of multiple cell signaling pathways. For example: the TGF-β/Smad signal pathway, which is important for the development of kidney fibrosis; when it is activated, the expression of MMP-2 and 9 are also upregulated [9]. The P38MAPK and Notch signal pathways are also very vital in the process of kidney fibrosis; some studies suggest that when they are activated, the expressions of MMP-2 and 9 are also upregulated [10,15,16] (Figure 1). With the aggravation of fibrosis, the renal tubular epithelial cells often exhibit hypoxia, and the hypoxia in turn also can lead to the upregulation of MMP-2 expression [17].

## 3. The Regulation of MMP-2 and MMP-9 Activity

### 3.1. The Basic Pathway of the Activity Regulation of MMP-2 and MMP-9

The translation product of MMPs’ mRNA is an inactive zymogen (pro-MMPs); when the propeptide region is cleaved, the pro-MMPs becomes activated and will become biologically active. The substances that can activate it are numerous, for example plasmin, which plays an important role in the process of renal interstitial fibrosis [18], and it is one of the important molecules to promote pro-MMPs’ activation [19]. Another example is urokinase-type plasminogen activator (UPA), whose upregulation is associated with the development of renal interstitial fibrosis [20], and it is also known to activate the pro-MMPs [21].

In addition to the above, tissue inhibitor of metalloproteinases (TIMPs) also has an important role in the activation of pro-MMPs; the TIMPs family includes four kinds of TIMPs (TIMP-1 to TIMP-4); TIMPs have a dual nature effect on MMPs like two sides of a coin: on the one hand, they can inhibit the activation of MMPs; on the other hand, they can promote the activation of MMPs. Take TIMP-2 as an example: it not only inhibits the activity of MMP-2, but it is also required for MMP-2 activation at a low concentration [22]. Membrane-type matrix metalloproteinases (MT-MMPs), one of the six sub-types of MMPs, also play a vital role in the activation of pro-MMPs. It is known that TIMP-2 mediation can lead to a combination of MT1-MMP (MMP-14) and pro-MMP-2 to form a complex-proMMP-2/TIMP-2/MT1-MMP on the membrane; thus, in this way, pro-MMP-2 can be activated as MMP-2 (Figure 1). Additionally, Toth et al. [23] showed that MT1-MMP and TIMP-2 also play a similar role in the activation of pro-MMP-9 to MMP-9. Meanwhile, MMP-2 also can promote the pro-MMP-9 to MMP-9 [24].

### 3.2. Effects of Cell Membrane Structural Change on MMP-2 and MMP-9 Activity

**Figure 2:**
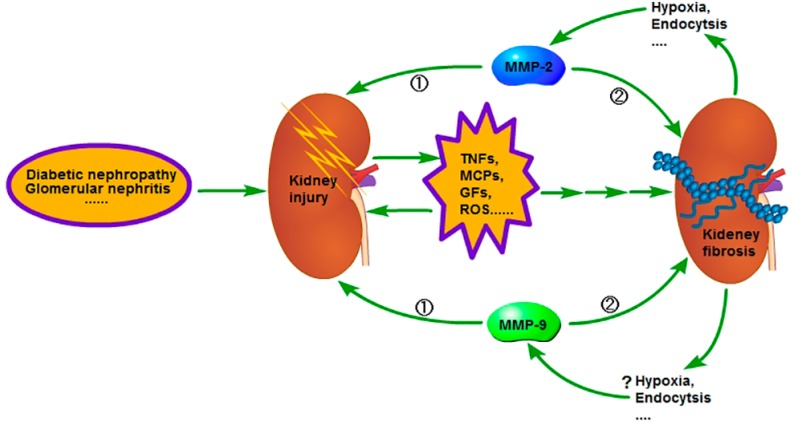
In the early stage of chronic kidney disease (CKD), when the kidney is injured, the injured cells and the inflammatory cells in kidney will secrete a variety of pro-inflammatory and pro-fibrotic cytokines, which promote the occurrence of renal interstitial fibrosis. Meanwhile, the activity of MMP-2 and 9 is increased and the renal basement membrane injured, promoting the phenotype transformation of renal tubular epithelial cells, at last resulting in the aggravation of extracellular matrix (ECM) deposition. However, in the advanced stage of CKD, the activity of MMP-2 and 9 is decreased and leads to inadequate degradation of ECM; therefore, the fibrosis is difficult to reverse. The reason for the activity decrease of MMP-2 in the advanced stage of CKD is related to the enhancement of endocytosis, which is caused by hypoxia, but further studies are needed regarding relation to the reason for the activity decrease of MMP-9 in the advanced stage of CKD. ① Early stage; ② advanced stage.

In the advanced stage of CKD, due to the excess formation of the renal fibrous septum, the renal interstitial capillary bed gets blocked, leading to further deterioration of the hypoxic state of renal tissue. Researches have already shown that [25] hypoxia can also decrease the activity of MMP-2 (Figure 2). Therefore, the interesting question would be why hypoxia can both increase the expression of MMP-2 and also reduce the activity of MMP-2. The answer is still not clear, yet the activation of MMP-2 has two pathways: the cell membrane pathway and the intracellular pathway; and studies have shown that the cell membrane pathway is the main one [26]; therefore, is it that the membrane structural change has the effect on it? Endocytosis is one of the important ways of changing the cell membrane structure [27], and under the hypoxic condition, endocytosis is enhanced [28,29]; so is it that endocytosis affects its activity? For caveolin protein-1 (caveolin-1), one of the key proteins involved in the endosome formation, research has shown that its interference in myocardial cells enhances the activity of MMP-2 [30], whereas its increased expression inhibits the activity of MMP-2 in HT1080 cells [31]. Recently, Yu et al. [32] have found that downregulation of its expression in the renal tubular epithelial cells can increase the activity of MMP-2. These studies suggest that enhanced endocytosis decreases MMP-2’s activity, but why? We mentioned above that MT1-MMP is important in the activation of MMP-2; however, the interaction between MT1-MMP and caveolin-1 can trigger the caveolin-1-dependent endocytosis of MT1-MMP [33,34], and Kim et al. [35] also found that caveolin-1 inhibits MT1-MMP activity. Therefore, endocytosis may be crucial in decreasing MMP-2 activity (Figure 1 and Figure 2). Therefore, does endocytosis have similar effects on MMP-9 activity? At present, there are no relevant research works, but during the process of MMP-9 activation, MT1-MMP also plays an important role, as previously mentioned; therefore it can be inferred that endocytosis may have similar effects on MMP-9 activity, as well (Figure 1 and Figure 2).

In addition to this, more than 10 years ago, scholars already discovered that [36,37] low density lipoprotein-related protein receptor 1 (LRP1) via the α2M proteasome can mediate the extracellular MMPs’ endocytosis, and by the interaction with the α2M proteasome, LRP1 can also regulate the distribution and activity of MT1-MMP, which is an another important pathway in the regulation of MMPs’ activity by endocytosis. Reversion-inducing cysteine-rich protein with kazal motifs (RECK) is also one of the important inhibitors of MMPs and MT-MMPs’ activity [38], but the specific mechanism is still unclear; whether it is possible that it is also related to endocytosis. Miki et al. [39] found that RECK could also regulate the endocytosis of MT1-MMP, and as MT1-MMP plays an important role in the activation of MMP-2 and 9, this suggested that endocytosis may be one of the important pathways for RECK in the regulation of MMP-2 and 9 activity (Figure 1). All of these findings suggest the importance of endocytosis in the regulation of MMPs’ activities. However, there are very few studies done on the regulation of MMPs’ activity by endocytosis, but its role cannot be ignored.

## 4. Role of MMP-2 and MMP-9 in Chronic Kidney Disease (CKD)

### 4.1. The Interaction between MMP-2, 9 and Tumor Necrosis Factors (TNFs) Promotes CKD Progression

TNF-α and TNF-β play an important role in the development of CKD [6,40]; what is the relationship between TNFs and MMPs and CKD? It has been proven that TNF-α can not only induce the expression of MMP-2 and 9 [41,42], but also promotes the activation of pro-MMP-2 [43] and thereby enhances the activity of MMP-2 [41,44]. Additionally, both TNF-α and TNF-β can promote the activation of pro-MMP-9 [45]. Conversely, if the activity of MMP-2 and 9 is inhibited, the process of converting pro-TNF-α to TNF-α will also be inhibited [46]. All of these studies suggest that TNFs interacts with MMPs (Figure 1). Therefore, these two are important in the occurrence and development of the progression of CKD.

### 4.2. The Effects of Interaction between MMP-2, 9 and Monocyte Chemoattractant Proteins (MCPs) on CKD

The MCPs family belongs to the chemokine CC subfamily; it includes five subtypes (MCP-1 to MCP-5), and it is known that MCP-1 and MCP-3 play an important role in the pathogenesis of renal interstitial fibrosis [7,47] (Figure 1). Some studies [48,49] have found that MCP-1 not only can simulate the expression of MMP-9, but also can enhance the activity of MMP-2, and the presence of MMP-2 can promote the degradation of MCP-3; thus, MCP-3 can be transformed into a common CC chemokine receptor antagonist, which inhibits the inflammatory response and the infiltration of mononuclear macrophages [50,51]. Therefore, in the absence of MMP-2, the degradation of MCP-3 may be reduced, which may promote the inflammatory response; thereby, a new hypothesis of the metalloproteinase/phospholipase A2 (SPLA2) axis is proposed [52] according to which the secretion of SPLA2 is increased when there is a lack of MMP-2 in the body. Additionally, as SPLA2 is important in inflammation, which promotes the inflammatory response, thus the activation of this axis will increases the related inflammatory response. Yet, SPLA2 also plays an important role in the development of renal injury and CKD [53,54]; therefore, in people with congenital MMP-2 deficiency, the occurrence and development progression of CKD may also be closely associated with this axis.

### 4.3. The Effects of Interaction between MMP-2, 9 and Growth Factors (GFs) on CKD

GFs is a factor of a large family including: epidermal growth factor (EGF), fibroblast growth factor (FGF), platelet-derived growth factor (PDGF), transforming growth factor (TGF), connective tissue growth factor (CTGF), etc. The contribution of different GFs in CKD is different. Some GFs promote the process of interstitial fibrosis, such as CTGF, TGF-β and EGF [55,56,57]; whereas some GFs may inhibit the process of renal interstitial fibrosis, such as hepatocyte growth factor (HGF), etc. [58]. Therefore, GFs seems to have an effect with a dual nature on the progression of CKD. Additionally, what is the specific relationship between GFs and MMP-2 and 9? For example, MMP-9 increases the expression of TGF-β and promotes the occurrence of renal interstitial fibrosis [59], while MMP-2 has an activation effect on FGF, which also promotes the development of fibrosis [60]. It was previously mentioned that HGF has an inhibitory effect on renal fibrosis, and the mechanism may be associated with the increased expression of MMP-2 and 9 [61].

Thus, there is an interesting problem in the process of development of CKD: some of the GFs promote its progression, whereas some others inhibit its development. The role of MMP-2 and 9 in the process of renal fibrosis is actually similar to that of GFs, such as: the activity of MMP-2 in the early stage of CKD is increased and can degrade the type IV collagen in renal basement membrane; injures the glomerular filtration membrane; promotes the upregulation of TGF-β; and promotes the renal tubular epithelial phenotype transformation; all of these promote the development of fibrosis [62]. However, at the advanced stage, the deposition of matrix is aggravated due to the inadequate activity of MMP-2 (Figure 2). The reason for the inadequacy of MMP-2 activity in the advanced stage is still unclear. As mentioned earlier, some studies did a preliminary investigation that one of the possible reasons could be due to renal interstitial fibrosis, which inhibits oxygen diffusion in the renal tubules, thereby enhancing endocytosis and the subsequent decrease of MMP-2’s activities. Studies [63] have found that the change of MMP-9 activity in renal interstitial fibrosis is similar to that of MMP-2, and the reasons for this are not clear; whether endocytosis plays an important role or not is yet to be elucidated (Figure 2).

### 4.4. The Effects of Interaction between MMP-2, 9 and Oxidative Stress on CKD

The role of oxidative stress (OS) in the pathogenesis of CKD is often important, and OS is often associated with neutrophils. The progression of CKD is associated with the degeneration and necrosis of the renal tissue; these cause the accumulation of neutrophils, which phagocytize this necrotic debris. During the process of phagocytosis by neutrophils, the oxygen consumption is increased, and part of the oxygen molecules under the catalytic reaction of NADPH and NADH oxidase can accept the extra electron and transform into oxygen free radicals, which induce OS [64]. Additionally, this further aggravates the inflammatory injury in CKD.

OS also can activate MMP-2 and 9 [65,66,67,68], the activity of which may change in different stage of CKD, as previously mentioned. With the progression of CKD, renal interstitial fibrosis is further aggravated, which further hinders the diffusion of oxygen, finally leading to serious hypoxia. The hypoxia further aggravates OS, thus forming a vicious cycle, which eventually leads to further exacerbation of fibrosis (Figure 2).

## 5. The Possible Relationship between MMP-2, 9 and Their Activating Molecule MMP-14 and Their Non-Proteolytic Functions with CKD

Recently, more and more attention has been given to the non-proteolytic functions of MMPs [69]. Therefore, in this part, we propose the possible relationship between non-proteolytic functions of MMP-2, 9 and their activation-related molecule MMP-14 with the occurrence and development of CKD.

### 5.1. The Possible Relationship between Non-Proteolytic Functions of MMP-2 and CKD

Many studies have been carried out about the role of the proteolytic functions of MMP-2 in the process of renal interstitial fibrosis. Therefore, what is the role of its non-proteolytic functions in the development of interstitial fibrosis? Some researchers have found that [70] in lung adenocarcinoma cells, pro-MMP-2 through the interaction with αVβ3 integrin can activate the phosphoinostitide 3-kinase/protein kinase B/hypoxia-inducible factor-1α (PI3K/AKT/HIF-1α) pathway. When this pathway is activated, the production of HIF-1α will increase. Additionally, HIF-1α can promote the expression of CTGF [71], and CTGF plays an important role in the development of CKD and renal interstitial fibrosis [72,73]. Therefore, in the process of the development of renal interstitial fibrosis, does pro-MMP-2 have a similar effect? If there is any, then this indicates that not only MMP-2, but also its precursor may have an important role in the initiation of a similar fibrotic pathway in the early stage; however, this claim needs to be confirmed by further studies.

### 5.2. The Possible Relationship between Non-Proteolytic Functions of MMP-9 and CKD

MMP-9 plays an important role in the occurrence and development of CKD. Studies have shown that MMP-9 also has non-proteolytic functions, for example [74], in Schwann cells, through the help of LRP1, MMP-9 can activate extracellular signal regulated kinase (ERK) 1/2. A similar function has not been reported in renal cells, but the activation of kinase is interlinked; related reports in Schwann cells stated that MMP-9 has the effect of ERK activation, and ERK has an important role in renal interstitial fibrosis [56]. Therefore, whether it is possible to inhibit the development of renal interstitial fibrosis by blocking these effects of MMP-9 warrants further study and discussions.

### 5.3. The Possible Relationship between Non-Proteolytic Functions of MMP-14 and CKD

It has been mentioned previously that with the mediation of TIMP-2, MMP-14 can be combined with pro-MMP-2 to form a complex of proMMP-2/TIMP-2/MT1-MMP on the cell membrane so that pro-MMP-2 can be activated to MMP-2. At present, the study [75] on MCF-7 breast cancer cells shows that the cytoplasmic tail of MMP-14 is required for the binding of TIMP-2 to MMP-14 on the MCF-7 surface, and when these two form as a complex, this can induce the proliferation and migration of MCF-7. What is more, in a mice xenograft model [75], an inactive proteolytic MMP-14 mutant still promotes the growth of tumor in mice; however, this effect was lost when its cytoplasmic tail was deleted. In another example [76], during the glycolysis in MMP-14 −/− macrophages, the adenosine triphosphate (ATP) concentration was reduced compared to before. This is because in macrophages, MMP-14 can stimulate glycolysis and the synthesis of ATP through its cytoplasmic tail effect. Therefore, in the early and advanced stages of renal interstitial fibrosis, if the abnormality of the MMP-14 cytoplasmic tail results in the abnormality of its non-proteolytic function, then can pro-MMP-2 still be successfully activated to MMP-2? If so, how does this affect the early and advanced stages of renal interstitial fibrosis? Will this aggravate it or alleviate it?

## 6. Conclusions

This review focuses on the regulation of gelatinases’ activity and their regulatory molecules in the development of CKD besides their role in the hydrolysis of ECM. The present studies show that gelatinases play a very important role in CKD, which is closely related to the multiple cell signaling pathways, endocytosis and the occurrence of renal inflammation in CKD. Additionally, their non-proteolytic functions may also be inextricably linked to the occurrence and development of CKD. Many studies on gelatinases focus on their traditional role of the hydrolysis of ECM, but in the meantime, we must also provide more emphasis on their other roles besides the traditional role. In this way, we can have a more specific and accurate understanding of their function in the human body. We hope that this can bring us more interesting discoveries.
